# *In vitro* sensitivity testing of minimally passaged and uncultured gliomas with TRAIL and/or chemotherapy drugs

**DOI:** 10.1038/sj.bjc.6604459

**Published:** 2008-07-01

**Authors:** D M Ashley, C D Riffkin, M M Lovric, T Mikeska, A Dobrovic, J A Maxwell, H S Friedman, K J Drummond, A H Kaye, H K Gan, T G Johns, C J Hawkins

**Affiliations:** 1 Children's Cancer Centre, Murdoch Children's Research Institute, Royal Children's Hospital, Parkville, Victoria 3052, Australia; 2 Department of Paediatrics, University of Melbourne, Parkville, Victoria 3010, Australia; 3 Department of Biochemistry, La Trobe University, Bundoora, Victoria 3086, Australia; 4 Department of Surgery, Duke University Medical Center, Durham, North Carolina 27710, USA; 5 Department of Surgery, University of Melbourne, The Royal Melbourne Hospital, Parkville, Victoria 3050, Australia; 6 Oncogenic Signalling Laboratory and Tumor Targeting Program, Ludwig Institute for Cancer Research, Heidelberg, Victoria 3084, Australia; 7 Department of Pathology, Molecular Pathology Research and Development Laboratory, Peter MacCallum Cancer Centre, Melbourne, Victoria 8006, Australia; 8 Department of Pathology, University of Melbourne, Parkville, Victoria 3010, Australia

**Keywords:** glioma, astrocytoma, glioblastoma, Apo-2L, apoptosis

## Abstract

TRAIL/Apo-2L has shown promise as an anti-glioma drug, based on investigations of TRAIL sensitivity in established glioma cell lines, but it is not known how accurately TRAIL signalling pathways of glioma cells *in vivo* are reproduced in these cell lines *in vitro*. To replicate as closely as possible the *in vivo* behaviour of malignant glioma cells, 17 early passage glioma cell lines and 5 freshly resected gliomas were exposed to TRAIL-based agents and/or chemotherapeutic drugs. Normal human hepatocytes and astrocytes and established glioma cell lines were also tested. Cross-linked TRAIL, but not soluble TRAIL, killed both normal cell types and cells from three tumours. Cells from only one glioma were killed by soluble TRAIL, although only inefficiently. High concentrations of cisplatin were lethal to glioma cells, hepatocytes and astrocytes. Isolated combinations of TRAIL and chemotherapy drugs were more toxic to particular gliomas than normal cells, but no combination was generally selective for glioma cells. This study highlights the widespread resistance of glioma cells to TRAIL-based agents, but suggests that a minority of high-grade glioma patients may benefit from particular combinations of TRAIL and chemotherapy drugs. *In vitro* sensitivity assays may help identify effective drug combinations for individual glioma patients.

The vast majority of malignant glioma patients die within 2 years of diagnosis, regardless of treatment (Group, 2002; Stupp *et al*, 2005). More effective treatments are therefore urgently required. Chemotherapy and irradiation trigger apoptosis of sensitive cells by provoking the ‘intrinsic’ apoptosis pathway (Norbury and Zhivotovsky, 2004). This involves the detection of DNA damage and instigation of a self-destruction program, which is regulated by the Bcl-2 family and executed by a molecular machinery including cytochrome c, Apaf-1 and the apoptotic proteases caspase-9 and caspase-3. Defects in the intrinsic pathway can contribute to resistance to chemotherapy and radiotherapy (Longley and Johnston, 2005). In contrast, ‘death ligands’, members of the TNF-*α* superfamily including FasL/CD95 and TRAIL/Apo2L, stimulate apoptosis through the ‘extrinsic’ pathway (Thorburn, 2004). Ligation of death receptors (such as Fas, DR4/TRAIL-R1 and DR5/TRAIL-R2) promotes recruitment of an adaptor molecule, FADD, caspase-8 and/or caspase-10 to form a complex known as the death-inducing signalling complex (DISC) (Kischkel *et al*, 1995). The initiator caspases are activated within the DISC, and acquire the ability to proteolytically activate effector caspases (such as caspase-3), either directly or indirectly (Scaffidi *et al*, 1998). The downstream caspases then destroy the cell by digesting numerous cellular proteins.

Because this extrinsic apoptosis pathway uses distinct components from that triggered by conventional anti-cancer treatments, there has been substantial research interest in exploiting its potential for treating tumour types that are unresponsive to currently available therapies. TRAIL and agonistic anti-TRAIL receptor antibodies are currently being evaluated in early-phase clinical trials. Initial reports portrayed TRAIL as an exemplary anti-cancer agent, as it induced apoptosis in many types of tumour cells but, unlike FasL, did not kill normal cells. Subsequent studies tempered that initial optimism somewhat. Although the extracellular portion of human TRAIL (amino acids 114–281, henceforth referred to as soluble TRAIL) was generally tolerated by normal human cells (Ashkenazi *et al*, 1999), other formulations were found to be toxic to particular normal cell types (Leverkus *et al*, 2000; Nitsch *et al*, 2000; Pettersen *et al*, 2002). Freshly isolated human hepatocytes displayed substantial sensitivity to His-tagged TRAIL, cross-linked TRAIL formulations and agonistic anti-DR4 and DR5 antibodies (Jo *et al*, 2000; Mori *et al*, 2004; Ganten *et al*, 2006), but survived incubation with soluble TRAIL (Ashkenazi *et al*, 1999; Lawrence *et al*, 2001; Ganten *et al*, 2006). The TRAIL sensitivity of normal brain cells is particularly relevant for the development of TRAIL-based anti-glioma therapies. Human astrocytes were relatively resistant to soluble untagged TRAIL *in vitro* (Ashkenazi *et al*, 1999; Song *et al*, 2006). Cell death was detected in brain slices incubated with FLAG-tagged TRAIL that had been cross-linked with an anti-FLAG antibody (Nesterov *et al*, 2002). Immunofluorescent assays suggested that the cells killed in these experiments included isolated neurons, oligodendrocytes, astrocytes and microglial cells (Nesterov *et al*, 2002).

Established glioma cell lines vary considerably in their responsiveness to TRAIL receptor ligation (reviewed in Hawkins, 2004). It is presently unknown how faithfully the death ligand signalling pathways of glioma cells *in vivo* are mimicked by established glioma cell lines, but it is has been shown that glioma cells do undergo substantial phenotypic changes *in vitro* (Anderson *et al*, 2002; Lee *et al*, 2006). TRAIL sensitivity of freshly resected uncultured glioma cells has not been reported to date. A few papers have documented the TRAIL responsiveness of minimally cultured gliomas, most of which were resistant to TRAIL as a sole agent (Roa *et al*, 2003; Song *et al*, 2003; Jeremias *et al*, 2004; Eramo *et al*, 2005; Li *et al*, 2006; Koschny *et al*, 2007). TRAIL can cooperate with other agents, including currently used chemotherapy drugs, to kill established glioma cells that survive exposure to TRAIL alone. It was recently published that the proteosome inhibitor bortezomib dramatically sensitised minimally passaged glioma cells to isoleucine-zipper-tagged TRAIL (Koschny *et al*, 2007). Importantly, however, the sensitivity of normal astrocytes to this co-treatment has not been reported.

Numerous mechanisms of glioma cell resistance to TRAIL have been suggested. Some resistant glioma cell lines could be sensitised by the treatment with the translation inhibitor cycloheximide, implicating a labile inhibitor of TRAIL signalling in the resistance of those cell lines (Rieger *et al*, 1998; Wu *et al*, 2000; Hao *et al*, 2001; Rohn *et al*, 2001; Fulda *et al*, 2002a). Lack of surface expression of TRAIL death receptors was reported in one glioma cell line (Arizono *et al*, 2003). Co-treatment of some resistant lines with chemotherapy drugs, which raised TRAIL receptor levels, enhanced TRAIL sensitivity (Nagane *et al*, 2000; Rohn *et al*, 2001; Shinohara *et al*, 2001; Arizono *et al*, 2003). Expression of inhibitors such as PKC*ε* (Shinohara *et al*, 2001), cFLIP (Hao *et al*, 2001; Xiao *et al*, 2002) or PEA-15 (Hao *et al*, 2001; Xiao *et al*, 2002) was associated with resistance in a small number of glioma cell lines, but causal relationships were not conclusively demonstrated. The ability of bortezomib to sensitise early passage glioma cells to TRAIL (Koschny *et al*, 2007) implies that proteosomal degradation of critical TRAIL pathway components could contribute to resistance in those cells. Inhibition of IAP activity in type II glioma cells sensitised them to TRAIL (Fulda *et al*, 2002b), indicating that IAP activity contributed to the TRAIL resistance of those lines. Low levels of caspase-8 may also contribute to TRAIL resistance (Knight *et al*, 2001; Ashley *et al*, 2005; Eramo *et al*, 2005).

A tenet underlying modern approaches to cancer treatment is that combination therapies can provide better selectivity and efficacy than single-agent treatments. To explore the possible clinical utility of combination TRAIL/chemotherapy treatment for malignant glioma, this study examined the responses of glioma cells, astrocytes and hepatocytes to TRAIL-based agents and/or chemotherapy drugs. Four TRAIL-related agents were tested: two forms of cross-linked TRAIL (F-LZ-TRAIL and ‘Superkiller’), the extracellular portion of TRAIL (‘soluble TRAIL’) and an agonistic anti-DR5 antibody. Seven chemotherapy drugs used for glioma therapy were also employed: cisplatin, carboplatin, CCNU, temozolomide, etoposide, vincristine and procarbazine. The drug combinations were tested on freshly resected gliomas and early passage glioma cell lines, to mimic as closely as possible the *in vivo* behaviour of malignant glioma cells.

## Materials and Methods

### Glioma samples and normal cells

Table 1 provides details about the patients whose tumours were assayed in this study. Gliomas RMH018-023 were resected at the Royal Melbourne Hospital, Australia. Informed consent was obtained from the patients, and approval for this study was obtained from the ethics committees of the Royal Children's Hospital, Royal Melbourne Hospital and La Trobe University. To generate a single cell suspension, tumour pieces were minced with a scalpel, then incubated with Accumax (Sigma, St Louis, MO, USA) and filtered through a tea stainer and 100 *μ*M filter. Viable cells were isolated by Ficoll density centrifugation.

The ‘D’ series of early passage lines was derived from specimens obtained from patients who had undergone tumour resection at Duke University Hospital (Durham, NC, USA). Informed consent was obtained from each patient prior to surgery in accordance with Duke Internal Review Board stipulations. The tumour material was collected in DMEM, 10% foetal bovine serum (FBS), 0.05 mg ml^−1^ gentamycin. Tumour samples were drained, placed in a 100 mm tissue culture dish and minced with sterile scissors. Warm sterile-filtered 0.4% collagenase solution (0.4% collagenase, 0.05 mg ml^−1^ gentamycin in zinc option-MEM, ZO-MEM) (Invitrogen, Carlsbad, CA, USA) was added to the minced tissue and incubated at 37°C for 1 h. Collagenase solution was inactivated by the addition of ZO-MEM, 10% FBS, 0.05 mg ml^−1^ gentamycin. Minced tissue was titurated to further homogenise sample and then centrifuged (1000 r.p.m., 5 min). Collagenase and media were removed, cells were re-suspended in fresh media and transferred to a 60-mm tissue culture dish and incubated at 37°C, 5% CO_2_. Cell cultures with a large RBC fraction were treated with haemolysis solution (0.83% ammonium chloride) as follows: cells were trypsinized and centrifuged (1000 r.p.m., 7 min), supernatant was discarded and cells were re-suspended in 2 ml FBC. Haemolysis solution was added at a ratio of 1 : 5 to 1 : 10. Haemolysis mixture was incubated at 4°C for 10 min, additional FBS was then added to bathe cells. Solution was centrifuged (1000 r.p.m., 7 min), following which the supernatant was discarded and the remaining cells were re-suspended in ZO-MEM, supplemented as described earlier. Tumour cultures were serially passaged using 0.25% trypsin-EDTA and collected in freezing medium (12.5% DMSO, 50% FBS and 37.5% ZO-MEM).

The early passage lines LM-G-2, LM-G-4 and LM-G-8 were made from tumours resected at the Austin Hospital (Heidelberg, VIC, Australia). Patients with known or suspected glioblastoma multiforme (GBM) were prospectively entered into a clinical trial after written informed consent was obtained. Ethics approval was granted by the Austin Hospital Human Research Ethics Committee. After intra-operative confirmation of a diagnosis of GBM, fresh tissue samples were obtained by biopsy or resection of tumour. Samples were mechanically disaggregated using the Medimachine (DAKO Diagnostika GmbH, Hamburg, Germany) (Brockhoff *et al*, 1999) and introduced into pre-warmed DMEM (Life Technologies, Grand Island, NY, USA) containing 10% FBS (CSL, Melbourne, VIC, Australia), 2 mM glutamine (Sigma Chemical Co, St Louis, MO, USA) and 50Uml^−1^ penicillin/ 50*μ*gml^−1^ streptomycin, respectively (Life Technologies). After 24 h, any non-adherent cells and material were discarded and the media replenished. Media were replenished twice weekly or more frequently if required. When cells appeared to have reached 50% confluence or maximal confluency in a T25 flask for that cell line, they were expanded into a T75 flask (passage 2). Cells were also expanded into a T75 (passage 3) flask when they reached 50% confluence or maximal confluency for that cell line. Thereafter, cells were expanded into T175 flask (passage 4) for cryostorage, experimentation or propagation as required.

Normal human hepatocytes and astrocytes were purchased from Cambrex (East Rutherford, NJ, USA). The established glioma cell lines D270 and U373 have been characterized previously (Knight *et al*, 2001, 2004). LN18 cells were purchased from the ATCC (Manassas, VA, USA).

### Drug treatments

We endeavoured to use physiologically relevant drug concentrations in this study. Cells were exposed to doses corresponding to 100% and 10% of peak plasma or tumour concentrations (Table 2). Data regarding the pharmacokinetics of the various TRAIL formulations in humans have not yet been published. We used soluble TRAIL (Peprotech, Rocky Hill, CT, USA) at concentrations commonly employed *in vitro* (1 *μ*g ml^−1^ and 100 ng ml^−1^). We arbitrarily chose to use F-LZ-TRAIL (Knight *et al*, 2001) at 10-fold lower doses than soluble TRAIL (100 and 10 ng ml^−1^), because our previous *in vitro* analyses showed that it is more potent than the untagged formulation (data not shown). Superkiller TRAIL (Alexis Biochemicals, Lausen, Switzerland) was used at 100 ng ml^−1^.

### Cell death/survival assays

During the experiments performed for this study, all cells were cultured in ZO-MEM supplemented with 10% FBS (SAFC Biosciences, Sydney, NSW, Australia). Cells were incubated with drugs for 48 h. The CellTiter-Glo kit (Promega, Madison, WI, USA) was used to quantitate survival, according to the manufacturer's instructions. Five hundred cells were used per treatment. Fifty thousand cells were used per condition for propidium exclusion assays (Knight *et al*, 2001), which were analysed using an LSRII (BD Biosciences, San Jose, CA, USA).

### Caspase activity assay

Ten thousand cells were incubated with normal media or TRAIL-based drugs in 96-well plates for 6 h, then caspase (DEVDase) activity was detected using the Caspase-Glo 3/7 kit (Promega), according to the manufacturer's instructions. Cell-specific luminescence signals were obtained by subtracting the signal generated from plates containing media or drugs but no cells from the signal obtained from wells containing cells and drugs.

### Immunoblotting

One hundred thousand cells were lysed, subjected to SDS–PAGE, immunoblotted and signals quantitated using previously published protocols (Ashley *et al*, 2005). The following antibodies were used: rabbit anti-DR4 and anti-DR5 from ProSci (San Diego, CA, USA) (no. 1139 and no. 2019, respectively), mouse anti-caspase-8 and anti-cFLIP from Alexis (Lausen, Switzerland) (clones 12F5 and NF6, respectively), mouse anti-FADD from BD Transduction Laboratories (San Jose, CA, USA) (clone 1/FADD), mouse anti-XIAP from MBL (Woburn, MA, USA) (clone 2F1), mouse anti-GAPDH from Chemicon (North Ryde, NSW, Australia) (clone 6C5), rabbit anti-p53 from Cell Signaling (Danvers, MA, USA) (no. 9282), goat anti-mouse-HRP (Sigma, no. A2304) and goat anti-rabbit-HRP (BD Biosciences no. 554021). Control lysates from 293T cells transiently transfected with plasmids directing the expression of caspase-8, FADD, cFLIP_L_ were generated as previously reported (Ashley *et al*, 2005). Similar control lysates were made using expression plasmids encoding XIAP, DR4 (kindly provided by Paul Ekert) and DR5. pIRES-PL-XIAP was synthesised as follows. Oligonucleotides 1 and 2 were annealed and ligated into pIRES-Neo (Clontech, Mountain View, CA, USA) cut with *Bam*HI and *Not*I, to yield pIRES-PL. The coding region of XIAP was amplified with primers 3 and 4, cut with *Eco*RI and *Not*I and ligated into *Eco*RI/*Not*I cut pIRES-PL, generating pIRES-PL-XIAP. pIRES-Neo-DR5 was made by amplifying the DR5 coding region with primers 5 and 6, cutting with *Eco*RI and *Bam*HI and ligating into pIRES-Neo (Clontech).

Oligonucleotides:

1: 5′-GGCCGAATTCGCGGGATCCGCGCGCTAGCAGCTGCGGCCGCAGGCCT-3′;
2: 5′-GATCAGGCCTGCGGCCGCAGCTGCTAGCGCGCGGATCCCGCGAATTC-3′;
3: 5′-GGAATTCCGCCATGACTTTTAACAGTTTTGAAGG-3′;
4: 5′-CCCCCGCGGCCGCTTAAGACATAAAAATTTTTTGCTTG-3′;
5: 5′-GGAATTCCGCCATGGAACAACGGGGACAG-3′; and
6: 5′-GCGGATCCTTAGGACATGGCAGAGTC-3′.

### p53 genotyping

Genomic DNA was extracted from frozen cell pellets (D2234MG, D2235MG, D2238MG, D2245MG, D2247MG, LM-G-4 and LM-G-8) or frozen cell suspensions (D2239MG, D2248MG, D2259MG, D2261MG, D2262MG and D2268MG, D2301MG) using the QIAamp DNA Blood Mini Kit (Qiagen, Hilden, Germany) according to the manufacturer's instructions. PCR cycling and high-resolution melt (HRM) analysis were performed on the Rotor-Gene 6000 (Corbett Research, Sydney, NSW, Australia). Each sample was analysed in triplicate. High-resolution melt analysis of exons 5–8 was performed as described previously (Krypuy *et al*, 2007). The amplicon of exon 4 (176 bp) covers the DNA binding domain and was generated using the primers TP53-Exon4-DBD-F, 5′-CCCCTGCACCAGCAGCTCCTA-3′ and TP53-Exon4-DBD-R, 5′-CAGCCCCTCAGGGCAACTGA-3′. The amplified region corresponds to GenBank accession number AC087388, nucleotides 78962–79137. PCR was performed in a 100 *μ*l PCR tube (Corbett Research) with a final volume of 20 *μ*l, containing 200 nmol l^−1^ of the forward primer, 300 nmol l^−1^ of the reverse primer, 200 *μ*mol l^−1^ of each dNTP, 0.5 U of HotStarTaq DNA Polymerase (Qiagen) in the supplied PCR buffer containing 2.0 mmol l^−1^ MgCl_2_, 5 *μ*mol l^−1^ SYTO9 (Invitrogen) and 2.5 ng of genomic DNA as template. The initial denaturation (95°C, 15 min) was followed by 11 cycles of 15 s at 95°C, 15 s at 65–60°C touchdown (0.5°C per cycle), 20 s at 72°C and 39 cycles of 15 s at 95°C, 15 s at 60°C, 20 s at 72°C; one cycle of 1 min at 95°C, 72°C for 1.5 min and a HRM step from 72 to 95°C rising at 0.2°C per second, and holding for 1 s after each stepwise increment. To confirm the mutation positive HRM results, PCR products of the entire exon 5 (exons 5a and 5b) and the HRM products of exons 6 and 8 were purified, directly sequenced in both directions and analysed as described previously (Krypuy *et al*, 2007).

## Results

### TRAIL sensitivity

**Figure 1:**
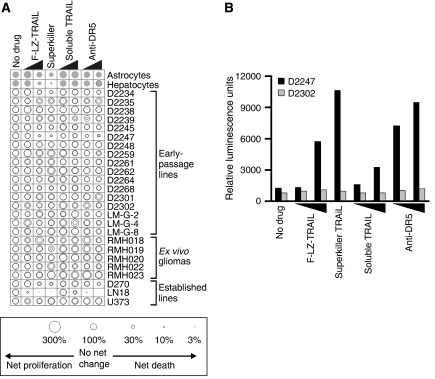
*In vitro* responses of glioma cells, astrocytes and hepatocytes to TRAIL. (**A**) Cells from the indicated early passage or established glioma cell lines, *ex vivo* gliomas, normal astrocytes or normal hepatocytes were incubated *in vitro* with TRAIL or with anti-DR5 antibody. Black triangles indicate high and low drug concentrations, when applicable (see Table 2 and the Materials and Methods section). Survival was assayed using the CellTiter Glo kit and depicted using ‘bubble’ graphs. The areas of the circles denote net survival following each treatment, relative to untreated cells (set at 100%, left column). Small circles indicate efficient killing, large circles reflect survival and/or proliferation, as illustrated in the graphical legend. Glioma assays were performed in duplicate (data are represented by circles). Four replicates were performed for hepatocytes and eight replicates for astrocytes. For astrocyte and hepatocyte data, grey circles depicting average survival are overlaid upon black circles indicating average survival plus standard error. (**B**) DEVDase activity in D2247 and D2302 cells was monitored 6 h following treatment with the specified TRAIL formulations, anti-DR5 antibody or normal media.

Cells from freshly resected gliomas, minimally passaged glioma cell lines, established glioma cell lines, normal astrocytes and hepatocytes were exposed to three formulations of TRAIL or an agonistic antibody, alone or in combination with seven chemotherapy drugs. Table 1 provides details of the glioma cells used and the patients from whom they were obtained. The normal cells tolerated exposure to ‘hepatosafe’ soluble TRAIL, the anti-DR5 antibody and the lower dose of cross-linked TRAIL (Figure 1A). Higher concentrations of cross-linked TRAIL and superkiller TRAIL were lethal to both types of normal cells, with hepatocytes being especially sensitive. As sole agents, the TRAIL formulations and anti-receptor antibody induced negligible cell death in most of the glioma samples tested. Only one of the early passage lines, D2247, was efficiently killed by the two cross-linked formulations of TRAIL and the anti-DR5 antibody. This line also displayed intermediate sensitivity to soluble TRAIL. Two other lines, D2234 and D2245, were somewhat sensitive to the cross-linked TRAIL formulations and the agonistic antibody, but not to soluble TRAIL. None of the *ex vivo* samples was substantially sensitive to any of the TRAIL-based treatments. As reported previously, LN18 and D270 were TRAIL-sensitive, but U373 was TRAIL-resistant (Hawkins, 2004). Consistent with the notion that apoptosis was responsible for the reductions in ATP levels observed in some drug-treated cells, caspase activity in D2247 cells but not in D2302 cells increased following exposure to TRAIL or anti-DR5 (Figure 1B).

### Cooperation between TRAIL and chemotherapy drugs

**Figure 2:**
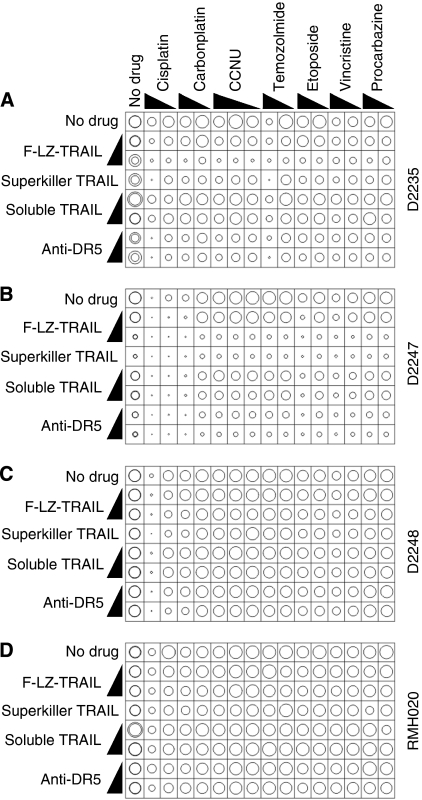
*In vitro* sensitivity of three early passage glioma cell lines and one *ex vivo* tumour to TRAIL in combination with chemotherapy drugs. Cells from the early passage lines D2235 (**A**), D2247 (**B**) and D2248 (**C**) and the freshly resected glioma RMH020 (**D**) were incubated *in vitro* with the stated formulations of TRAIL or anti-DR5 antibody, alone or together with the listed chemotherapy drugs as described in the Materials and Methods section and Table 2. The resulting survival was assayed and graphed as described in the legend to Figure 1.

**Figure 3:**
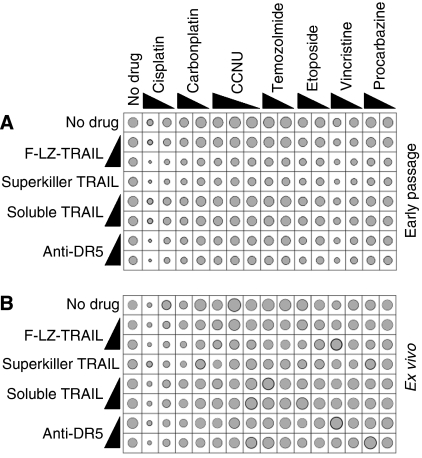
*In vitro* sensitivity of glioma cells to TRAIL in combination with chemotherapy drugs. Cells from 17 early passage glioma cell lines (**A**) and 5 uncultured gliomas (**B**) were incubated *in vitro* with the stated formulations of TRAIL or anti-DR5 antibody, alone or together with the listed chemotherapy drugs. The resulting survival was assayed and graphed as described in the legend to Figure 1. Grey circles depicting average survival are overlaid upon black circles indicating average survival plus standard error.

**Figure 4:**
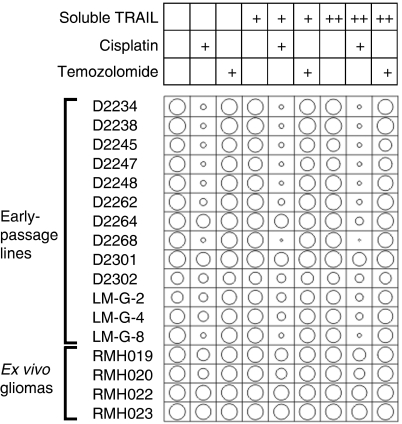
Propidium iodide uptake assay of glioma cell sensitivity to combination treatments. Cells from the indicated early passage glioma cell lines or *ex vivo* gliomas were incubated *in vitro* with soluble TRAIL at 100 ng ml^−1^ (+) or 1000 ng ml^−1^ (++) alone or together with temozolomide (13.7 *μ*g ml^−1^) or cisplatin (54 *μ*g ml^−1^) for 48 h. Flow cytometry measurement of propidium iodide exclusion was used to quantitate the proportion of surviving cells. The areas of the circles denote survival following each treatment. Small circles indicate efficient killing, large circles reflect survival.

Previous studies have shown that co-treatment with traditional anti-cancer agents can sensitise some cells to TRAIL, *in vitro* and *in vivo*. We tested the ability of the various TRAIL formulations to kill glioma cells in conjunction with a number of chemotherapy drugs used in glioma therapy (Table 2). Figure 2 illustrates the effect of these combination treatments on D2247 (the most TRAIL-sensitive line), D2235 and D2248 (two of the TRAIL resistant lines) and RMH020 (one of the uncultured tumours). Average responses across all early passage lines and *ex vivo* tumours are shown in Figure 3. Many samples were killed by high-dose cisplatin, however a 10-fold lower concentration was much less effective. The higher dose of vincristine was weakly toxic to many of the glioma samples. Only the D2235 cells were markedly sensitive to temozolomide. The other drugs were ineffective as sole agents. All chemotherapy drugs tested further sensitised D2247 to F-LZ-TRAIL and anti-DR5 (Figure 2), but only additive toxicity was observed when TRAIL-resistant cells were exposed to the combination treatments (Figures 2 and 3 and data not shown). The possibility that prior treatment with chemotherapy drugs may enhance sensitivity to TRAIL was also explored. Cells from a TRAIL-resistant early passage line, D2302, were incubated with chemotherapy drugs only for 24 h, then TRAIL or anti-DR5 antibody was added for an additional 48 h period. These treatments had similar effects on cellular ATP levels to co-incubations with TRAIL plus chemotherapy drugs for either 48 or 72 h (Supplementary Figure 1), indicating that prior exposure to chemotherapy drugs did not sensitise D2302 cells to TRAIL. Propidium iodide uptake assays were performed on many of the samples, using selected drug combinations. This method, which gives a direct measure of the proportion of cells killed, yielded similar data to the CellTiter-Glo assay, which quantifies cellular ATP (Figure 4).

### Normal cells

**Figure 5:**
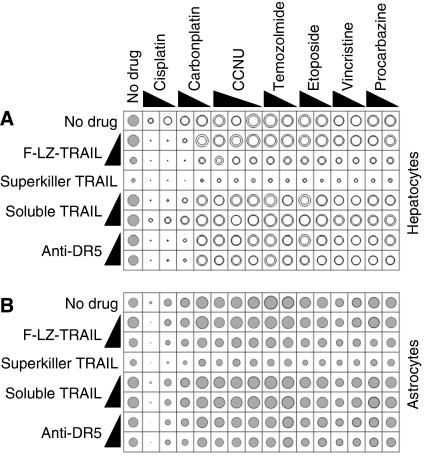
*In vitro* sensitivity of astrocytes and hepatocytes to TRAIL in combination with chemotherapy drugs. Normal human hepatocytes (**A**) and astrocytes (**B**) were incubated *in vitro* with the stated formulations of TRAIL or anti-DR5 antibody, alone or together with the listed chemotherapy drugs. The resulting survival was assayed and graphed as described in the legend to Figure 1. (**A**) Quadruplicate assays were performed to examine hepatocyte survival following incubation with each TRAIL formulation alone. Grey circles depicting average survival are overlaid upon black circles indicating average survival plus standard error. Responses to combination treatment were assayed in duplicate; circles depict each result. (**B**) Eight replicates were performed to investigate astrocyte survival following incubation with each TRAIL formulation alone. Combination treatments were tested in quadruplicate. Grey circles depicting average survival are overlaid upon black circles indicating average survival plus standard error.

As previously published (Ashkenazi *et al*, 1999; Jo *et al*, 2000; Lawrence *et al*, 2001; Mori *et al*, 2004; Ganten *et al*, 2006), normal human hepatocytes were sensitive to cross-linked TRAIL formulations, but not to soluble untagged TRAIL (Figure 1). Hepatocytes were also efficiently killed by cisplatin and carboplatin (Figure 5). In general, chemotherapy drugs did not further sensitise hepatocytes to TRAIL, although treating hepatocytes with high-dose F-LZ-TRAIL and the chemotherapy drugs elicited a slightly superadditive effect (Figure 5). Normal human astrocytes were also sensitive to cross-linked TRAIL, cisplatin and carboplatin, but not to the same extent as hepatocytes. No significant cooperation in astrocyte lethality was noted between TRAIL and the chemotherapy drugs (Figure 5).

**Figure 6:**
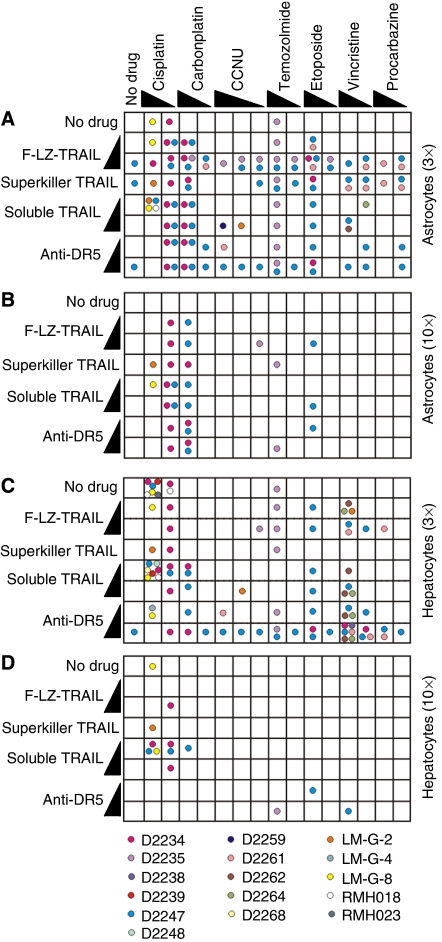
Treatments selectively toxic *in vitro* to glioma cells relative to normal cells. Coloured circles indicate gliomas killed *in vitro* by each treatment at least 3 times (**A**, **C**) or 10 times (**B**, **D**) more efficiently than the most sensitive astrocyte replicate (**A**, **B**) or hepatocyte replicate (**C**, **D**).

On average, the minimally cultured and uncultured glioma cells were as sensitive or less sensitive than the normal cells to TRAIL-based drugs, alone or in combination with chemotherapeutic agents. However, isolated examples of selective toxicity to glioma cells relative to normal cells were observed (Figure 6). TRAIL formulations combined with platinum-based drugs killed cells from a few gliomas (D2234, D2247, LM-G-8 and LM-G-2) at least 10 times more efficiently than normal astrocytes and hepatocytes. Temozolomide cooperated with TRAIL to kill D2235 cells more efficiently than the normal cells. Some selectivity of TRAIL/etoposide and TRAIL/vincristine combinations was observed for D2247 relative to normal cells.

### Pathway analyses

**Figure 7:**
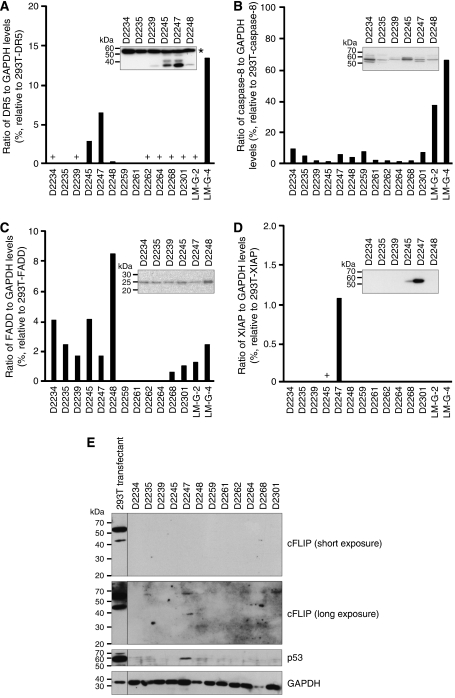
Immunoblot analyses of candidate TRAIL signalling regulators. Immunoblotting was performed on lysates from the indicated glioma early passage cell lines or 293T cells transiently transfected with expression plasmids encoding the various apoptotic pathway components. (**A**–**D**) Anti-DR5 (**A**), anti-caspase-8 (**B**), anti-FADD (**C**) and anti-XIAP (**D**) signals were quantitated using a chemidoc instrument and plotted relative to the 293T transfectant lysates (set at 100%). A nonspecific band detected by the anti-DR5 antibody is indicated by an asterisk. Long exposures using autoradiograph film revealed some signals too weak to be detected by chemidoc (+). Illustrative immunoblots are inset within each graph. (**E**) Autoradiography was used to assay cFLIP_L_ and p53 expressions in early passage lines and 293T cells transfected with the cFLIP_L_ expression plasmid (cFLIP_L_, GAPDH immunoblots) or empty vector (p53 immunoblot). Irrelevant lanes separating the 293T transfectant signals from those of the early passage lines have been removed.

To explore potential mechanisms underlying the resistance of most of the gliomas to TRAIL-induced apoptosis, we surveyed minimally passaged gliomas for the expression of the TRAIL pathway components DR4 (TRAIL-R1), DR5 (TRAIL-R2), FADD and caspase-8, along with potential modulators of TRAIL signalling (cFLIP and XIAP) (Figure 7A–7D). None of the lines expressed detectable DR4 (data not shown) or cFLIP_S_ (Figure 7E). Expression of the other components varied widely between samples. Four of the early passage lines that were TRAIL-resistant (D2259, D2261, D2262 and D2264) did not express detectable FADD. The TRAIL-sensitive line D2247 expressed relatively high levels of DR5 and XIAP and detectable, if relatively low, levels of FADD, caspase-8 and cFLIP_L_. D2235 and D2301 also expressed low levels of cFLIP_L_. The p53 status of the majority of lines was also examined. Mutations that may affect function were identified in four of the lines (D2234, D2235, LM-G-4 and LM-G-8) (Supplementary data). D2247 was the only line of those tested to express sufficient p53 to detect by immunoblotting (Figure 7E).

## Discussion

Two conditions would have to be met for TRAIL to be clinically effective for treating malignant glioma: (a) a route of administration must be used that delivers concentrations of TRAIL that are lethal to the patient's glioma cells *in vivo* and (b) the glioma cells must be markedly more sensitive to TRAIL than normal cells exposed via that mode of delivery. Any treatment for brain tumours must transverse or bypass the blood–brain barrier. This means that any future TRAIL-based therapies for glioma would probably be administered intracranially. Intracranial delivery may also lessen hepatocyte exposure and thus reduce hepatotoxicity. Multiple intracranial delivery systems are being developed. The post-resection tumour cavity can be lined with drug-impregnated wafers (Westphal *et al*, 2006). Convection-enhanced delivery is a promising new technique in which drugs are infused at the tumour site under pressure, thus improving distribution into the mass of the tumour (Lopez *et al*, 2006). Mice bearing intracranial glioma xenografts were successfully treated with TRAIL administered using this approach (Saito *et al*, 2004).

Numerous studies have concluded that TRAIL is a promising anti-glioma drug based on the investigation of TRAIL sensitivity and signalling in established glioma cell lines. However, it has been reported that melanoma cells exhibited enhanced TRAIL sensitivity following *in vitro* culture (Nguyen *et al*, 2001). To minimise the potential for *in vitro* culturing artefacts, in this study we tested the TRAIL sensitivity of minimally cultured and freshly resected gliomas. Our analyses imply that minimally passaged and uncultured gliomas respond similarly to TRAIL. It is, however, possible that prolonged *in vitro* culturing, as with the commonly studied established glioma lines, may significantly affect TRAIL sensitivity.

Minimally cultured and uncultured glioma cells were generally resistant to the TRAIL formulations tested. None of the tumours tested was efficiently killed by the ‘hepatosafe’ formulation of TRAIL currently being evaluated in early-phase clinical trials. Three of the gliomas were sensitive to cross-linked formulations of TRAIL: D2234, D2245 and D2247. Intriguingly, the patients from whose tumours those lines were derived had all received chemotherapy before surgery. In contrast, only one patient whose tumour was TRAIL-resistant received treatment prior to resection (RMH020). Unfortunately, normal astrocytes and hepatocytes were also sensitive to cross-linked TRAIL. Only one glioma (D2247) was killed by cross-linked TRAIL more efficiently than normal astrocytes.

Four of the early passage lines lacked detectable FADD, possibly accounting for their TRAIL resistance. The TRAIL-sensitive line D2247 bore only low levels of FADD and caspase-8, arguing that low concentrations of these pathway components can be sufficient for TRAIL-induced apoptotic signalling. D2247 was one of a number of lines lacking mutations in p53, and the only line tested to express p53 levels detectable by immunoblotting. Consistent with this observation, D2247 also expressed high levels of DR5, a known p53-inducible protein (Wu *et al*, 1997). LM-G-4, a TRAIL resistant line, expressed higher levels of DR5, caspase-8 and FADD than D2247, arguing that factors other than the levels of these proteins influence TRAIL sensitivity in glioma cells. The expression of the potential TRAIL inhibitors XIAP and cFLIP was higher in the TRAIL-sensitive D2247 cells than the resistant lines, arguing against overexpression of these proteins as a mechanism of resistance in gliomas that survived incubation with TRAIL. Definition of the molecular mechanisms contributing to the resistance of the majority of the gliomas to TRAIL will require additional investigation, but it seems unlikely that a single resistance mechanism, perhaps amenable to therapeutic manipulation, will be found to account for the widespread survival of glioma cells following exposure to TRAIL.

Better therapies are urgently needed for malignant glioma. *In vitro* sensitivity assays could be used for preclinical evaluation of the anti-glioma potential of new drugs. In the future, such assays could also assist in the selection of the most effective drug combinations for individual patients. Our data imply that few glioma patients would benefit from TRAIL-based therapies; perhaps *in vitro* sensitivity testing could help identify the minority of patients most likely to respond. The luminescence assay used in this study is rapid, high-throughput and requires fewer cells than the commonly employed MTT and flow cytometric assays, thus allowing more drug doses and combinations to be tested per sample. The accuracy with which *in vitro* sensitivity testing predicts *in vivo* responses may be influenced by the mechanism of action of the particular drug as well as the degree to which cellular environment influences the toxicity of each agent to glioma cells. For example, glioma cell interactions with surrounding cells and the extracellular matrix could modulate the apoptosis-inducing capacity of anti-cancer drugs *in vivo*. For these reasons, it is also important to examine orthotopic models of cancer. Notwithstanding these considerations, *in vitro* apoptosis data do tend to correlate with patient responses in cancer types for which truly effective drugs exist (Nagourney, 2006). The dearth of effective drugs for treating glioma has limited assessment of the predictive value of *in vitro* sensitivity testing for this tumour type. Nevertheless, available evidence does argue that *in vitro* sensitivity testing can assist in selecting treatments for glioblastoma patients (Iwadate *et al*, 2003).

Currently, the majority of glioblastoma patients receive surgical resection, radiotherapy and temozolomide. Although temozolomide may benefit some patients, it is well recognised that the majority of patients do not respond to this drug. Temozolomide administration only extended median survival of glioblastoma patients from 12.1 to 14.6 months post-diagnosis, boosting 2-year survival from 10.4 to 26.5% (Stupp *et al*, 2005). Given this subtle effect *in vivo*, it is perhaps not surprising that only one of the tumours tested in this study (D2235) was efficiently killed by temozolomide *in vitro*. Cisplatin was the most effective of the drugs tested *in vitro*, however strong toxicity was triggered only by the higher of the doses employed, which corresponded to the peak intratumoral cisplatin level following embolisation (Tegeder *et al*, 2003). Only partial sensitivity was observed using a 10-fold lower concentration, which resembles peak tumour and plasma levels achieved following systemic cisplatin administration (Riva *et al*, 2000; Tegeder *et al*, 2003; Watanabe *et al*, 2003; Siegel-Lakhai *et al*, 2005). A few gliomas were more sensitive than normal cells to co-treatments with TRAIL and chemotherapy drugs.

In conclusion, our data indicate that TRAIL-based therapies would be unlikely to benefit the majority of glioma patients. This study does, however, suggest that particular patients may respond to specific combinations of TRAIL and chemotherapy drugs. *In vitro* sensitivity assays may prove useful in identifying such patients and predicting effective drug combinations.

## Figures and Tables

**Table 1:** Patient and cell line features

**Tumour/ cell line**	**Sex, age (years)**	**Tumour grade (WHO)**	**Passage number**	**p53 genotype^a^**	**Treatment before sample obtained**	**Treatment after sample obtained**	**Progression free survival**	**Patient status**
D2234	M, 52	IV	10	M	Radiotherapy, temozolomide	BCNU wafer, O6BG, cloretazine, AP23573	2 months	Died 7 months post-resection
D2235	F, 20	III	9	M	Nil	Radiotherapy, temozolomide, CCNU, tamoxifen	35+months	Stable 35 months post-resection
D2238	F, 31	III	7	W	Nil	Radiotherapy, temozolomide, CCNU, tamoxifen	34+months	Stable 34 months post-diagnosis
D2239	M, 56	IV	7	W	Nil	Radiotherapy, temozolomide, hydroxyurea, imatinib mesylate	3 months	Died 4 months post-resection
D2245	M, 44	III	9	W	Temozolomide	Radiotherapy, CCNU, tamoxifen, imatinib mesylate, hydroxyurea, CCNU, bevacizumab, CPT-11	18 months	Stable 32 months post-resection
D2247	M, 51	IV	8	W	Radiotherapy, temozolomide	BCNU wafer, O6BG, CPT-11, imatinib mesylate, hydroxyurea	4 months	Died 9 months post-resection
D2248	M, 44	III	6	W	Nil	Unknown	Data not available	Data not available
D2259	M, 27	III	7	W	Nil	Radiotherapy, temozolomide, CCNU	31+months	Stable 31 months post-resection
D2261	M, 52	III	5	W	Nil	Radiotherapy, temozolomide, CCNU, cloretazine	7 months	Died 10 months post-resection
D2262	F, 40	III	5	W	Nil	Radiotherapy, temozolomide	3 months	Unknown
D2264	F, 45	IV	4	ND	Nil	Radiotherapy, temozolomide, CCNU, CPT-11, tamoxifen	30+months	Stable 30 months post-resection
D2268	M, 59	IV	6	M^1^	Nil	Radiotherapy, temozolomide, CCNU, CPT-11, imatinib mesylate, hydroxyurea/PTK787, bevacizumab, carboplatin	7 months	Stable 26 months post-resection
D2301	M, 51	IV	1	M^2^	Nil	BCNU wafer, radiotherapy, temozolomide, cilengitide, CCNU, imatinib mesylate, hydroxyurea, PTK787	3 months	Stable 13 months post-resection
D2302	M, 40	IV	3	ND	Nil	Radiotherapy, temozolomide, etoposide, re-resection	24+months	Stable 24 months post-resection
LM-G-2	M, 54	IV	4	ND	Nil	Sub-total resection then radiotherapy	4+months	Alive 4 months post-resection
LM-G-4	M, 69	IV	3	M	Nil	Gross total resection then radiotherapy	12 months	Died 20 months post-resection
LM-G-8	M, 74	IV	3	M	Nil	Radiotherapy	2 months	Died 5 months post-resection
RMH								
018	M, 54	III	*ex vivo*	ND	Nil	Recurrence, resection	data not available	Alive 6 months post-resection
RMH								
019	M, 62	IV	*ex vivo*	ND	Nil	Radiotherapy and temozolomide	data not available	Alive 6 months post-resection
RMH								
020	F, 52	IV	*ex vivo*	ND	Resections, radiotherapy, temozolomide	Chemotherapy, radiotherapy	data not available	Alive 6 months post-resection
RMH								
021	M, 66	IV	*ex vivo*	ND	Nil	Radiotherapy and temozolomide	data not available	Alive 4 months post-resection
RMH								
022	F, 54	IV	*ex vivo*	ND	Nil	Radiotherapy	data not available	Died 3 months post-resection
RMH								
023	M, 58	IV	*ex vivo*	ND	Nil	Radiotherapy and temozolomide	data not available	Alive 4 months post-resection but recurrence

HRM=high-resolution melt; M=mutation possibly affecting p53 function; ND=not done; WT=wild type; M^1^=silent mutation; M^2^=mutation predicted by HRM analysis but not identified by sequencing. For details see Supplementary Figure 1.

a p53 genotype, as determined by HRM analysis and sequencing.

**Table 2:** Chemotherapy drugs and doses used in this study

**Drug**	**Concentrations used in this study**	**Published human peak intratumour or plasma concentrations**
Soluble TRAIL	1 *μ*g ml^−1^ 100 ng ml^−1^	Not reported
F-LZ-TRAIL	100 ng ml^−1^ 10 ng ml^−1^	Not reported
Superkiller TRAIL	100 ng ml^−1^	Not reported
Anti-DR5	3 *μ*g ml^−1^ 0.3 *μ*g ml^−1^	Not reported
Cisplatin	54 *μ*g ml^−1^ 5.4 *μ*g ml^−1^	Peak tumour concentration after embolisation was 54 *μ*g ml^−1^, after perfusion was 11.4 *μ*g ml^−1^ (Tegeder *et al*, 2003). Peak plasma concentrations ranged from 1.5 *μ*g ml^−1^ (Riva *et al*, 2000; Urien *et al*, 2005) to around 4 *μ*g ml^−1^ (Siegel-Lakhai *et al*, 2005; Watanabe *et al*, 2003)
Carboplatin	44 *μ*g ml^−1^ 4.4 *μ*g ml^−1^	Peak glioma concentration was 13 *μ*g ml^−1^, peak plasma concentration was 44 *μ*g ml^−1^ (Whittle *et al*, 1999)
CCNU	9 *μ*g ml^−1^ 900 ng ml^−1^ 90 ng ml^−1^	Peak plasma concentration of active metabolites was reported to be 9 *μ*g ml^−1^ (Lee *et al*, 1985) or 1–2 *μ*g ml^−1^ (Kastrissios *et al*, 1996)
Temozolomide	13.7 *μ*g ml^−1^ 1.37 *μ*g ml^−1^	Peak plasma concentration was 13.7 *μ*g ml^−1^ (Brada *et al*, 1999)
Etoposide	10.5 *μ*g ml^−1^ 1.05 *μ*g ml^−1^	Peak tumour concentration was 1.04–4.80 *μ*g g^−1^. Peak plasma concentration was 7–10.5 *μ*g ml^−1^ (Kiya *et al*, 1992)
Vincristine	40.4 ng ml^−1^ 4 ng ml^−1^	Peak plasma concentration was 40.5 ng ml^−1^ but rapidly decreased to 5 ng ml^−1^ (Groninger *et al*, 2005)
Procarbazine	540 ng ml^−1^ 54 ng ml^−1^	Peak plasma concentration was 540 ng ml^−1^ (He *et al*, 2004)
