# *In vitro* sensitivity testing of minimally passaged and uncultured gliomas with TRAIL and/or chemotherapy drugs

**DOI:** 10.1038/sj.bjc.6604990

**Published:** 2009-03-17

**Authors:** D M Ashley, C D Riffkin, M M Lovric, T Mikeska, A Dobrovic, J A Maxwell, H S Friedman, K J Drummond, A H Kaye, H K Gan, T G Johns, C J Hawkins

**Correction to**: British Journal of Cancer (2008) **99**, 294–304; doi:10.1038/sj.bjc.6604459

On correction of the above article, the affiliations of some of the authors were incorrectly presented. The correct affiliations are as follows:

DM Ashley^1,2^, CD Riffkin^1^, MM Lovric^3^, T Mikeska^4^, A Dobrovic^4,5^, JA Maxwell^6^, HS Friedman^6^, KJ Drummond^7^, AH Kaye^7^, HK Gan^8^, TG Johns^8^ and CJ Hawkins^*^^,1,2,3^

^1^Children's Cancer Centre, Murdoch Children's Research Institute, Royal Children's Hospital, Parkville 3052, Australia; ^2^Department of Paediatrics, University of Melbourne, Parkville 3010, Australia; ^3^Department of Biochemistry, La Trobe University, Bundoora 3086, Australia; ^4^Molecular Pathology Research and Development Laboratory, Department of Pathology, Peter MacCallum Cancer Centre, Melbourne, Victoria 8006, Australia; ^5^Department of Pathology, University of Melbourne, Parkville, Victoria 3010, Australia; ^6^Department of Surgery, Duke University Medical Center, Durham 27710, USA; ^7^Department of Surgery, University of Melbourne, The Royal Melbourne Hospital, Parkville 3050, Australia; ^8^Oncogenic Signalling Laboratory and Tumor Targeting Program, Ludwig Institute for Cancer Research, Heidelberg 3084, Australia

