# Supplementary material for: In vitro sensitivity testing of minimally passaged and uncultured gliomas with TRAIL and/or chemotherapy drugs
Source: Br J Cancer. 2008 Jul 1;99(2):294–304. doi: 10.1038/sj.bjc.6604459 (PMC2480982; doi:10.1038/sj.bjc.6604459)
Supplement: Supplementary Figure 1 and Data [file 6604459x1.pdf]

Supplementary Figure 1

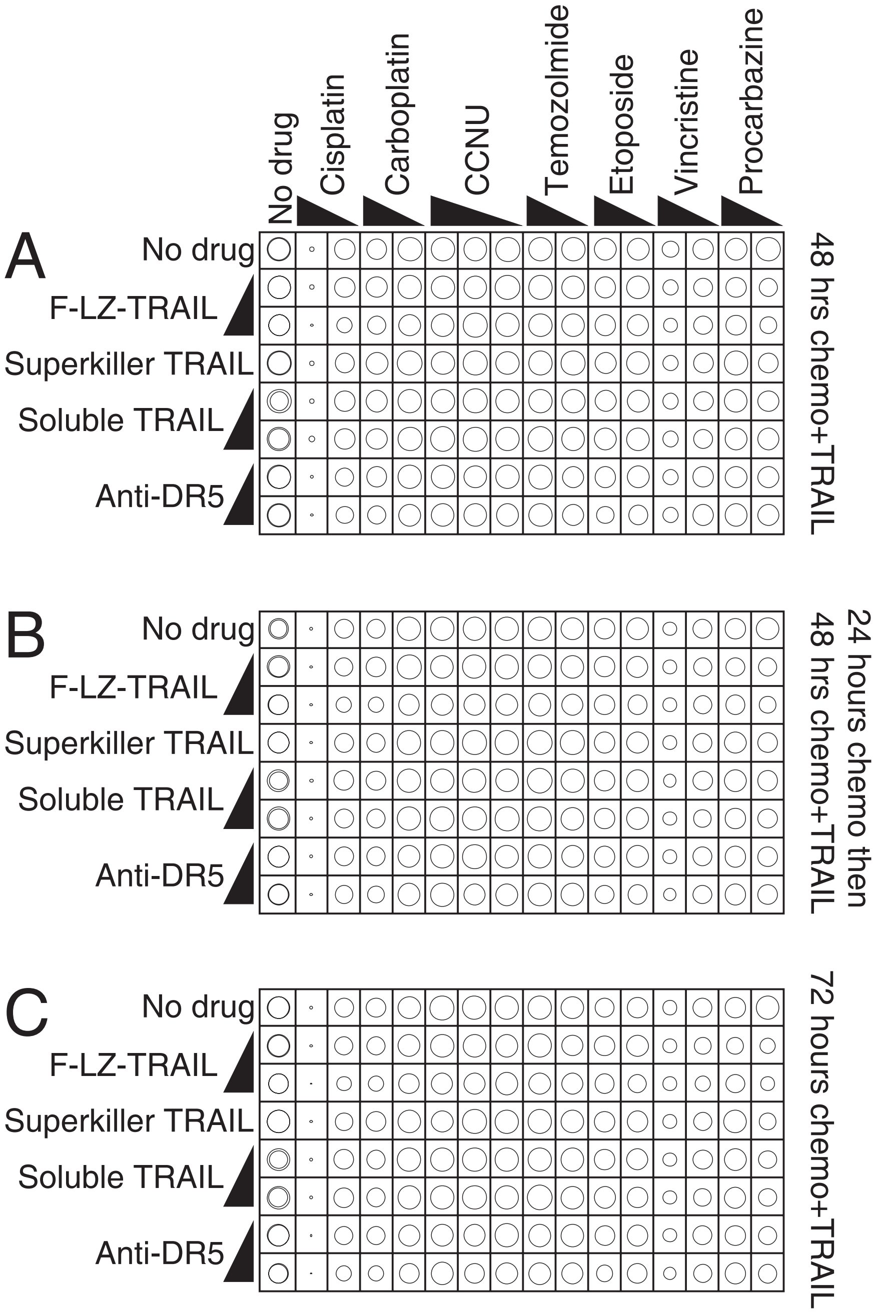

Prior incubation with chemotherapy drugs does not sensitize D2302 cells to TRAIL. Cells from the early passage line D2302 were incubated *in vitro* with the stated formulations of TRAIL or anti-DR5 antibody, alone or together with the listed chemotherapy drugs as described in the Materials and Methods section. The resulting survival was assayed and graphed as described in the legend to Figure 1. (A) Cells were incubated simultaneously with the specified drug combinations for 48 hours. (B) Cells were incubated with the chemotherapy drugs alone for 24 hours, then the TRAIL-based agents were added and the cells incubated for a further 48 hours. (C) Cells were incubated simultaneously with the specified drug combinations for 72 hours.

Supplementary Data

p53 status of early passage glioma cell lines.

Fourteen cell lines (D2234, D2235, D2238, D2239, D2245MG, D2247, D2248, D2259, D2261, D2262, D2268, D2301, LM-G-4 and LM-G-8) were screened for mutations in exons 4 to 8 of TP53 by high resolution melting (HRM), as outlined in the Materials and Methods section. The regions screened encode the DNA binding domain of p53. Mutations in the DNA binding domain contribute to p53 dysfunction in many cancers (Vogelstein, B. et al., Nature, 408, 307, 2000). The HRM data for D2234, D2235, D2268, D2301, LM-G-4 and LM-G-8 indicated clear mutations.

Direct sequencing of the mutation positive HRM products was performed, as described in the Materials and Methods section. D2268 was heterozygous for the synonymous and therefore non-pathogenic SNP, 639A>G (R213R), (rs1800372) in exon 6. Two cell strains, D2234 and D2235 showed a homozygous or hemizygous change in intron 5 (IVS5-9T>A). It is unclear whether this change is pathogenic. LM-G-8 showed a heterozygous mutation in exon 8, 840A>T, which substitutes the polar, basic arginine by the polar, neutral serine (R280S). LM-G-4 was homozygous or hemizygous for another mutation in exon 8, 844C>T R282W, which results in an amino acid exchange of the polar, basic arginine by the non-polar, neutral tryptophan.
